# Dexamethasone on absent end-diastolic flow in umbilical artery, in growth restricted fetuses from early-onset preeclamptic pregnancies and the perinatal outcome

**DOI:** 10.1080/07853890.2021.1968030

**Published:** 2021-08-31

**Authors:** Oana Sorina Tica, Andrei Adrian Tica, Doriana Cojocaru, Mihaela Gheonea, Irina Tica, Dragos Ovidiu Alexandru, Victor Cojocaru, Lucian Cristian Petcu, Vlad Iustin Tica

**Affiliations:** a Department of “Mother and Child”, University of Medicine and Pharmacy of Craiova, Craiova, Romania; b Emergency Clinical County Hospital of Craiova, Craiova, Romania; c Department of Pharmacology, University of Medicine and Pharmacy of Craiova, Craiova, Romania; d Department of Anesthesiology and Intensive Care, Nicolae Testemitanu State University of Medicine and Pharmacy Chisinau, Chisinau, Moldova; e Timofei Mosneaga Republican Clinical Hospital, Chisinau, Moldova; f Department of Pediatrics, University of Medicine and Pharmacy of Craiova, Craiova, Romania; g Department of Internal Medicine, Faculty of Medicine, University “Ovidius” Constanta, Constanta, Romania; h University Regional Emergency Hospital of Constanta, Constanta, Romania; i Department of Biostatistics, University of Medicine and Pharmacy of Craiova, Craiova, Romania; j Department of Biophysics, Faculty of Dental Medicine, University “Ovidius” Constanta, Constanta, Romania; k Department of Obstetrics and Gynecology, Faculty of Medicine, University “Ovidius” Constanta, Constanta, Romania

**Keywords:** Early onset preeclampsia with severe features, growth restriction, absent end-diastolic flow, umbilical artery, dexamethasone, perinatal outcome

## Abstract

**Background:**

Absent end-diastolic flow (AEDF) in the umbilical artery (UA) worsens the already poor prognosis of growth-restricted fetuses (GRFs) in pregnancies complicated by early-onset preeclampsia with severe features (ESP).

**Method:**

We assessed the correlation between the effect of maternal dexamethasone (Dex) on AEDF in the UA and perinatal outcomes, in 59 GRFs from EPS-complicated pregnancies. The maternal outcome was also evaluated.

**Results:**

The mean maternal age at inclusion was 22.4 ± 5.9 years. Dex transiently restored EDF in the UA in 38 (64.4%) cases (trAEDF group), but in 21 (35.6%) patients, the flow was persistently absent (prAEDF group). The effect lasted up to the 4th day.

The gestational age at diagnosis, number of days from admission until delivery, and fetal weight were significantly lower in the prAEDF group than in the trAEDF group (*p* < .05). The same group had a significantly increased rate of fetal proximal deterioration, low APGAR scores, neonatal hypoxia, assisted ventilation, mild intraventricular haemorrhage (I/II), and respiratory distress syndrome, as well as maternal deterioration, especially in cases of resistant hypertension (*p* < .05). Although the rates of fetal acidemia and perinatal mortality in the prAEDF group were respectively three times and two times higher, the differences were not significant (*p* > .05).

**Conclusions:**

The Dex no-effect on UA Doppler in GRFs with AEDF in the UA, in EPS-complicated pregnancies, can be a useful marker for a higher risk of proximal fetal deterioration, poor state at delivery, neonatal hypoxic complications, and worsening maternal condition, but not for perinatal mortality. The findings also highlight the alarmingly younger age of patients with EPS. Finally, all these pregnancies should be monitored in a complex multidisciplinary manner in tertiary referral units.

**Key message**
- The effect of dexamethasone on absent end-diastolic flow in the umbilical artery in growth-restricted fetuses from pregnancies complicated by early-onset preeclampsia with severe features can be a useful prognostic factor for perinatal outcomes.

## Introduction

Preeclampsia remains a serious and high-risk complication of pregnancy for both the mother and child. It affects approximately 2–8% of pregnancies and is continuously increasing in frequency or severity [1–3]. In a 20-year study (1980–2000), Ananth [1] reported a mild increase in the frequency of preeclampsia from 3.4% to 3.8% in the United States. However, the incidence of severe forms increased from 0.3% to 1.4%, representing a relative increase of 322%.

The pathogenesis of preeclampsia is still not completely understood, but many reports have linked it to defective placentation, which causes maternal discharge of several antiangiogenic and proinflammatory substances that induce characteristic systemic endothelial dysfunction [2–4]. This disease remains a major cause of maternal mortality, with over 50,000 deaths worldwide each year [5]. The consequences for the fetus are low blood flow in the umbilical circulation, followed by fetal growth restriction (FGR) and prenatal death [2,3,6].

Absent end-diastolic flow (AEDF) or reversed end-diastolic flow (REDF) in the umbilical artery (UA) are Doppler aspects of FGR that occur later as a result of increased resistance in the umbilical circulation [7].

Preeclampsia is one of the most common causes of preterm newborns, accounting for 25–43% of preterm births, and steroids are widely used to improve fetal outcomes during prematurity [1,8,9].

Our study focussed on the predictive value of the effect induced by maternal administration of dexamethasone (Dex) on AEDF in the UA of growth restricted fetuses (GRFs) in pregnancies complicated by early-onset preeclampsia with severe features (EPS), with respect to perinatal outcomes. Maternal outcomes were also quantified.

## Study design

The study was performed between January 2008 and December 2020 at three tertiary referral care units: the University County Hospital of Craiova, Romania; the University Regional Emergency Hospital of Constanta, Romania; and the Timofei Mosneaga Republican Clinical Hospital of Chisinau, Moldova. The inclusion criteria for the study group were pregnancy complicated with EPS, FGR, and AEDF in the UA.

The common criteria for the diagnosis of EPS included systolic blood pressure >160 mmHg and diastolic pressure >120 mmHg at one measurement, or >110 mHg at two measurements after 6 h of bed rest, occurring between 20 and 34 weeks of gestation in a previously healthy woman, in association with proteinuria (defined as ≥300 mg/day). In cases with no proteinuria, the diagnosis of EPS, in addition to hypertension, was based on one of the following features of severity: oliguria (<500 mL/day), thrombocytopenia (<100,000/mm^3^), pulmonary edema, newly appearing persistent headaches or scotoma, newly persistent right upper quadrant pain or epigastric pain, liver enzyme levels more than twice the normal blood values, or newly occurring and progressive renal insufficiency [10,11].

We based the diagnosis of FGR or intrauterine growth restriction (IUGR) on the Fetal Medicine Foundation’s recommendation: fetal abdominal circumference (AC) below the 5^th^ percentile of the normal range for the respective gestational age (GA) [12] and severe FGR below the 3^th^ percentile.

Maternal status and history were carefully scrutinised. Exclusion criteria were diabetes mellitus, obesity, vascular and/or connective tissue disorders, concomitant steroid treatment for other reasons, smoking and/or alcohol consumption during pregnancy, multifetal pregnancies, and genetic or non-genetic fetal malformations.

All patients received similar medications for their hypertensive disorders. Magnesium sulphate was used to prevent seizures and provide neonatal neuroprotection.

Corticosteroid therapy followed the institutional protocols for preterm births. This therapy consisted of four doses of 6 mg Dex administered intramuscularly every 12 h.

UA Doppler evaluation was performed prior to Dex administration (day 0, hour 0) and at 24, 48, 72, and 96 h after the first dose. The patients who delivered within the first 48 h after the first administration were discharged from the study, as we considered the period too short for satisfactory evaluation of the effect of Dex.

In addition to the UA, Doppler was used to assess the middle cerebral artery (MCA) and ductus venosus (DV) for optimal fetal monitoring and to enable more appropriate decision-making at the time of delivery.

We did not include fetuses with REDF in the UA and/or those associated with abnormal DV Doppler. For these cases, our institutional protocols recommend rapid delivery after (if possible) steroid therapy. This approach is concordant with the report of a mortality rate as high as 70% in REDF fetuses [13] and an increased risk of intrauterine death at any gestational age in the absence or reversed A-wave on DV Doppler [14].

After delivery, all placentas were sent to the pathology laboratory (as a rule for all births performed in our medical units). In addition, as per institutional guidelines, because all fetuses included in the present study were at high risk of neonatal complications, arterial cord blood samples were sent to the laboratory for analysis.

Finally, the perinatal and supplementary maternal outcomes were corroborated with the effect of steroids on UA Doppler waveforms.

The patients were informed about the study design and agreed for their and their babies’ medical data to be anonymized used for academic purposes.

All numerical data were statistically analysed using a one-way analysis of variance (one-way ANOVA test). Statistical significance was set at *p* < .05, and a value of *p* < .001 was considered highly significant. Odds ratios (ORs) were calculated to assess the influence of end-diastolic flow modifications induced by Dex on different outcome indicators. The accepted confidence interval (CI) was 95%.

## Results

Between January 2008 and December 2020, 151,257 births were registered at the three medical institutions. After applying the inclusion criteria, we selected 71 patients from the study group. Twelve pregnant women had delivered within 48 h of the first dose of Dex and were discharged. Ultimately, the study group consisted of 59 patients.

Under maternal Dex administration, in 38 (64.4%) cases, the EDF in the UA transiently returned to the positive level. These patients comprised the transiently restored (absent) end-diastolic flow (trAEDF) group. The remaining 21 (35.6%) fetuses showed no variation and comprised the persistently-absent end-diastolic flow group (prAEDF). When present, the effect started on the first day of corticoid treatment, was maximum on the second day, and could last until day 4.

The average age of the patients was 22.4 ± 5.9 years (range, 15–41 years), with similar values in both groups (Table 1). The GA at inclusion was calculated according to the first day of the last menstrual period (GA [LMP]) and was confirmed in some cases using a first-trimester ultrasound examination. GA was significantly lower in the prAEDF group (Table 1).

**Table 1.** Maternal and fetal characteristics at admission.

	prAEDF group (*n* = 21)	trAEDF group (*n* = 38)	Statistics	
			*p*	Test’s value
Age (years)				
Mean ± SD	22.8 ± 6.2	22.2 ± 5.7	.717	*f-r* = 0.131
Interval	15–39	16–41	---	---
GA(LMP) (weeks)				
Mean ± SD	28w4d ± 8d	29w6d ± 9d	<.001	*f-r* = 13.582
Interval	26w2d–30w6d	27w4d–32w4d	---	---
FGR				
<3th percentile	21 (100%)	38 (100%)	---	---

prAEDF/trAEDF: persistent/transiently restored absent end-diastolic flow in umbilical artery; GA(LMP): gestation age after last menstrual period; w: weeks; d: days; FGR: fetal growth restriction; SD = standard deviation; *f-r: f*-ratio of one-way ANOVA test; *p* < .05 was considered statistically significant.

All fetuses with AEDF in the UA were severely growth-restricted (Table 1).

The rate of fetal deterioration, defined by spontaneous heart rate decelerations and/or worsening Doppler was significantly higher in the prAEDF group than in the trAEDF group. This was mainly due to the higher incidence of the more severe ultrasound aspects (newly occurred reversed EDF in UA and/or absence or reversal of A-wave in DV) (Table 2).

**Table 2.** Delivery route and the rate of spontaneous and induced labour. Total

Delivery route	prAEDF (21)	trAEDF (38)	Statistics	
			*p*	OR (95%CI)
C-section	16 (76.2 %)	28 (73.6 %)	.832	1.1 (0.3–3.9)
Fetal interest				
Fetal deterioration				
Bradycardia	3 (14.2%)	3 (7.8%)	.442	1.9 (0.3–10.6)
Worsening Doppler	10 (47.6%)	6 (16.7%)	.011	4.8 (1.4–16.4)
Total	11 (52.3 %)**	8 (22.8 %)**	.016	4.1 (1.2–13.1)
Labour <32 weeks (prematurity)	3 (14.2 %)	7 (18.4 %)	.685	0.7 (0.1–3.2)
Maternal interest				
Worsening general maternal condition*				
Uncontrolled AHT	10 (47.6 %)	8 (22.8 %)	.038	3.4 (1.0–10.8)
Ascites	2 (9.5 %)	2 (5.2 %)	.538	1.8 (0.2–14.5)
Renal dysfunction	2 (9.5 %)	2 (5.2 %)	.538	1.8 (0.2–14.5)
Total	12 (57.1 %)**	11 (28.9 %)**	.007	4.4 (1.5–13.4)
Eclampsia*	3 (14.2 %)	5 (13.1 %)	.903	1.1 (0.2–5.1)
Abruptio placentae*	0 (0.0 %)	1 (2.6 %)	.743	0.5 (0.02–14.9)
Retinal detachment*	1 (4.7 %)	2 (5.2 %)	.993	0.9 (0.1–10.5)
HELLP sd.*	3 (14.2 %)	6 (15.7 %)	.877	0.8 (0.1–3.9)
Vaginally	5 (23.8 %)	10 (26.3 %)	.832	0.8 (0.2–3.0)
Labour				
Spontaneous labour	5 (23.8 %)	14 (36.8 %)	.308	0.5 (0.1–1.7)
Induced labour***	3 (14.2 %)	3 (7.9 %)	.442	1.9 (0.3–10.6)
Total	8 (38.1 %)	17 (44.7 %)	.621	0.7 (0.1–2.2)

prAEDF/trAEDF: persistent absent/transiently restored absent end-diastolic flow; AHT: arterial hypertension; *: events occurred at least 48 h after dexamethasone administration; **: there were events’ association; ***: for fetal death *in utero*; *f-r* =* f*-ratio of one-way ANOVA test; OR (95%CI): Odds ratio 95% confidence interval; *p* < .05 was considered statistically significant.

There were three intrauterine fetal deaths (IUFDs) in each group, and the difference was not statistically significant. All six dead fetuses were delivered vaginally after induction of labour (Tables 2 and 4).

**Table 4.** The neonatal mortality in the two groups.

Group		Causes		GA(LMP) at delivery (weeks)		Fetal weight at delivery (g)		Days from			
								Admission		Delivery	
prAEDF											
IUFD		I. FD		27		780		6		---	
3/21		II. SIUD		27		740		3		---	
(14.2 %)		III. SIUD		29		860		5		---	
Neonatal		I. Extreme prematurity*		26		520		---		2	
4/18		II. SPC + IVH III/IV		29		870		---		6	
(22.2 %)		III. Multiple morbidities*		30		850		---		4	
		IV. Sepsis		31		850		---		9	
Total 7/21 (33.3 %)											
trAEDF											
IUFD		I. FD		29		880		6		---	
3/38		II. Abruptio placentae		29		910		7		---	
(7.9 %)		III. FD		31		1040		4		---	
Neonatal		I. Sepsis		29		890		---		8	
5/35		II. SPC		29		920		---		6	
(14.2 %)		III. Multiple morbidities*		30		1010		---		5	
		IV. Sepsis		30		1120		---		11	
		V. Multiple morbidities		31		1190		---		7	
Total 8/38 (21.0 %)											

IUFD: intrauterine fetal death; FD: fetal deterioration; SIUD: sudden intrauterine unexpected death; SPC: severe pulmonary complications; IVH: intraventricular haemorrhage; GA(LMP): gestational age after last menstrual period; prAEDF/trAEDF: persistent/transiently restored absent end-diastolic flow in umbilical artery; *revealed metabolic acidosis at birth.

The spontaneous labour rate was higher in the trAEDF group than in the prAEDF group, but this increase was not statistically significant (Table 2).

The prAEDF group had a significantly shorter time from inclusion to imposition of delivery (“days gained”) (Table 3).

**Table 3.** The perinatal outcome in the two groups.

Fetal outcome	prAEDV (18*)	trAEDF (35*)	Statistics	
			*p*	Test’s value
GA(LMP) (at delivery) (weeks)				
Mean ± SD	29w4d ± 9d	30w5d ± 8d	.002	*f-r* = 9.928
Interval	26w3d–31w4d	28w4d–33w1d	–	–
Birth weight (g)				
Mean ± SD	885 ± 108	1.074 ± 112	<.001	*f-r* = 33.161
Interval	520–1.030	850–1.340	–-	–
Days gained from admission*				
Mean ± SD	5.1 ± 1.3	6.8 ± 2.3	.009	*f-r* = 7.272
Interval	(2–7)	(3–11)	–	–-
APGAR score				
At 1 min < 6	12 (66.6 %)	11 (31.4 %)	.017	OR: 4.3 (1.2–14.6)
At 5 min < 6	9 (50.0 %)	5 (14.2 %)	.007	OR: 6.0 (1.6–22.5)
RDS	10 (55.5 %)	8 (22.8 %)	.020	OR: 4.2 (1.2 − 14.2)
IVH				
I/II	9 (50.0 %)	6 (17.1 %)	.015	OR: 4.7 (1.3 − 17.3)
III/IV	1 (5.5 %)	0 (0 %)	.276	OR: 6.0 (0.2 − 157.1)
ROP (I/II)	6 (33.3 %)	7 (20.0 %)	.289	OR: 2.0 (0.5 − 7.2)
PVL	1** (5.5 %)	0 (0 %)	.276	OR: 6.0 (0.2 − 157.1)
Sepsis	5 (27.7 %)	6 (17.1 %)	.369	OR: 1.8 (0.4 − 7.2)
Perinatal hypoxia	18 (100 %)	15 (42.8 %)	.008	OR: 48.9 (2.7 − 876.5)
Fetal metabolic acidemia	3 (16.6%)	2 (5.7%)	.215	OR: 3.3 (0.4 − 21.8)
Hypoglycemia	2 (11.1 %)	3 (8.5 %)	.765	OR: 1.3 (0.3 − 10.6)
Hypotension	12 (66.6 %)	15 (42.8 %)	.105	OR: 2.6 (0.8 − 8.7)
Thrombocytopenia	6 (33.3 %)	8 (22.8 %)	.415	OR: 1.6 (0.4 − 5.9)
Perinatal mortality				
IUFD	3/21 (14.2 %)	3/38 (7.9 %)	.442	OR: 1.9 (0.3 − 10.6)
Neonatal death	4/18 (22.2 %)	5/35 (14.2 %)	.350	OR: 2.0 (0.4 − 8.5)
Total	7/21 (33.3%)	8/38 (21.0 %)	.303	OR: 1.8 (0.5 − 6.2)
NICU admission	12 (100 %)	23 (100 %)	–	–
Assisted ventilation				
Nb. neonates	8 (66.6 %)	9 (25.7 %)	.005	OR: 5.6 (1.6 − 19.9)
Nb. days / newborn				
(Mean ± SD)	3.1 ± 1.16	1.3 ± 0.47	<.001	*f-r* = 20.666
Interval	(1 − 5)	(1 − 2)	–-	–-
Days in O_2_ (mean)				
Mean ± SD	24.6 ± 11.5	18.7 ± 7.9	.036	*f-r* = 4.463
Interval	(13 − 36)	(7 − 37)	–-	–-
Hospitalisation (days)				
Mean ± SD	51 ± 26.3	48 ± 19.9	.540	*f-r* = 0.380
Interval	(39 − 74)	(26 − 75)	–-	–-

prAEDF/trAEDF: persistent/transiently restored absent end-diastolic flow in umbilical artery; *: live births (3 stillborn in each group); RDS: respiratory distress syndrome; IVH: intraventricular haemorrhage, mild/moderate forms – I/II, respectively severe forms III/IV; ROP (I/II): retinopathy of prematurity, mild/moderate forms I/II; PVL: periventricular leukomalacia; IUFD: intrauterine fetal death, **associated porencephaly and developed cerebral palsy; NICU: neonatal intensive care unit; SD: standard deviation; w: weeks; d: days; *f-r* =* f*-ratio of one-way ANOVA test; OR: (): Odds ratio (95% confidence interval); *p* < .05 was considered statistically significant.

The rate of worsening of general maternal condition, especially with respect to uncontrolled arterial hypertension (AHT), was significantly increased in the prAEDF group. Severe maternal incidents, including eclampsia, retinal detachment, and HELLP syndrome, occurred at a similar frequency in both groups. There was one case of placental abruption in the trAEDF group. The fetus died *in utero* (Table 2). No maternal deaths were observed.

Our institutional guidelines mandate Caesarean section (C-section) for all live fetuses under 32 weeks of GA(LMP), and optional C-section for those between 32 and 34 weeks (after 34 weeks, vaginal delivery is recommended). In the present study, all births occurred before 34 weeks of GA; thus, conforming to the institutional guidelines, and based on indications for fetal and/or maternal severe distress, we chose C-section for all patients. However, in nine patients (two in the prAEDF group and seven in the trAEDF group), vaginal delivery was performed because labour was too advanced at the time of examination. Finally, the decisions for choosing a C-section were almost similarly proportional between the two groups. (The reasons for performing C-sections are detailed in Table 2).

After delivery, all live neonates were admitted to the neonatal intensive care unit (NICU).

Newborns from the prAEDF group displayed significantly lower GA(LMP) and fetal weights, and higher rates of small APGAR scores (at 1 min and especially at 5 min), compared with newborns in the trAEDF group (Table 3).

Hypoxia, defined as an oxygen saturation of ˂ 30% in fetal arterial blood [15], was observed in all neonates in the prAEDF group, but in only 42.8% of fetuses belonging to the trAEDF group, a statistically significant difference. Consecutively, the newborns from the first group required a higher rate and longer period of assisted ventilation as well as a longer period of supplementary oxygen (Table 3).

Three cases from the prAEDF group and two from the trAEDF group met the criteria for fetal metabolic acidosis, defined as pH < 7.1 and a base deficit of ≥12 mEg/L in the fetal arterial blood [16]. The differences were not statistically significant. In these cases, the morbidity was 100%, with a neonatal mortality of up to 66.6% (2/3) and 50% (1/2) in the prAEDF and the trAEDF groups, respectively (Table 4).

The rate of respiratory distress syndrome (RDS) and mild intraventricular haemorrhage (grade I/II – IVH I/II) occurred significantly more frequently in the prAEDF group than in the trAEDF group. Severe IVH (grade III/IV) was detected in one neonate. Another patient was diagnosed with periventricular leukomalacia (PVL), associated porencephaly, and later developed cerebral palsy. Both newborns were from the prAEDF group (Table 3).

The incidence of sepsis, retinopathy of prematurity (ROP) grades I and II, thrombocytopenia, hypoglycemia, and hypotension were not significantly increased in the prAEDF group compared to the trAEDF group (Table 3).

Dex had no effect on maternal circulation.

With respect to the placental evaluation, there were no significant differences in either the incidence or severity of specific preeclamptic structural abnormalities between the two groups.

Neonatal mortality was not significantly increased in the prAEDF group when compared with the trAEDF group. The same assertion was correct for total perinatal mortality as well (Table 3).

## Discussion

The significantly reduced GA(LMP) at admission in the prAEDF group compared with the trAEDF group resulted from an earlier diagnosis of EPS in the first group. This was due to the earlier occurrence of maternal symptoms such as hypertension, headache, edema, abdominal pain, and visual disturbance. Moldenhauser [17] reported that placental abnormalities were more severe when the disease occurred during early gestation. However, although it was very probable that earlier symptoms were the result of more severe systemic maternal damage, we found no structural differences between placentas of both groups.

It seems that absent or reversed EDF in the UA occurs when 60% to 70% of the villous vasculature is affected [18,19]. Thus, it was not surprising that in our study group, all fetuses with absent EDF in the UA secondary to significantly decreased umbilical flow, were severely growth-restricted.

Maternal administration of Dex was followed by a transient return from absent to positive EDF in 64.1% of cases. Muller [20], Robertson [21], and Ekin [22] reported similar reversal rates of end-diastolic UA flow in growth-restricted fetuses of any etiology. The consensus is that there is no difference between the efficacies of Dex and betamethasone, with selection depending only on clinical experience [23,24]. However, Senat [25] and Mulder [26] reported a higher impact on the fetal variability of heart rate and cerebral circulation with betamethasone than with Dex.

The mechanism of transient restoration from absent (or reversed) EDF to positive EDF in the UA is unknown. It may be mediated by placental corticotrophin-releasing hormone (CRH) [26]. Exogenous administration of corticosteroids decreases maternal hypothalamic CRH secretion but significantly increases placental production [27]. The peptide binds specific CRH-R2 receptors in the placenta and in the umbilical circulation, with increased expression of nitric oxide synthase (NOS), followed by increased nitric oxide (NO) production and intense vasodilatation [28]. The “selective” effect of corticosteroids on umbilical arteries is due to the little (or no) evidence of the presence of CRH-R2 in the utero-placental circulation [29]. However, during the adult disease state (as seen in ESP), in the systemic circulation, steroids can directly and non-transcriptionally activate endothelial NOS (eNOS) and modulate its activity in a high-dose brief-exposure manner [30]. This phenomenon may be responsible for maternal improvement in some cases and can contribute to the improvement of fetal circulation and consecutively, fetal oxygenation. Another suggested mechanism consists of steroid-induced increased expression of membrane K^+^ channels in the small intravillous arteries, with vasodilation and decreased resistance [31].

The Dex-related effect was most strongly correlated with the rate of hypoxia at birth. All fetuses in the prAEDF group were hypoxic compared with less than half in the trAEDF group. Thus, there was probably an increased incidence of antenatal hypoxia in the first group leading to a significantly higher rate of antenatal fetal deterioration, which was especially revealed by the worsening Doppler.

Along with the increased rate of hypoxia, the lack of effect of corticosteroids on UA Doppler was a good predictor of poorer state at birth: higher rates of low APGAR scores, higher rates and longer periods of assisted ventilation, and more days of supplementary oxygen.

Paradoxically, although fetal metabolic acidemia (a direct consequence of hypoxia [32]) had a three-fold increase in incidence in the prAEDF group compared with the trAEDF group, the difference was not statistically significant, probably due to the limited number of cases.

The occurrence of RDS and mild IVH (I/II) paralleled the ratio between the rates of hypoxia in the prAEDF group compared with those in the trAEDF group (2.5- to 3-fold increase). This provides proof of their hypoxic pathophysiology. The Dex effect on the UA showed no correlation with IVH (III/IV) and PVL, with only one case of each registered.

Sepsis, which is a major cause of perinatal morbidity and mortality in newborns, especially premature newborns, and prolonged hospitalisation [33], occurred only at a slightly increased rate in the prAEDF group compared with the trAEDF group, despite the significantly higher prematurity in the first group. The similar rates were the result of long admission periods in both groups.

The incidence of mild and moderate ROP (I/II), fetal hypotension, hypoglycemia, and thrombocytopenia paralleled the neonate’s grade of prematurity, with a slight and non-significant augmentation in the prAEDF group.

There are several controversial reports on the prognostic value of A(R)EDF in the UA for neonatal complications in restricted fetuses, but all analysed the GRFs by any etiology, in association or not to hypertensive disorders [13,34–41].

The total perinatal mortality was 33.3% in the prAEDF and 21.0% in the trAEDF groups, respectively, but this difference was not statistically significant. A similar incidence of perinatal mortality in fetuses with AEDF was reported by Karstrup (28%) [13] and Eronen (30%) [34]. We found that neonatal mortality was dependent on prematurity, with 22.2% mortality in the prAEDF group at a mean GA(LMP) of 29w4d ± 11 days, and 14.2% mortality in the trAEDF group at a mean GA(LMP) of 30w5d ± 8 days. For EPS, Hall [42] reported neonatal mortality rates of 28% at 28 weeks and 8% at 29 weeks of gestation.

Mortality in neonates was increased by 50% in the prAEDF group but IUFD was twice as high in the same group, when compared with the trAEDF group (14.2% vs. 7.9%). Although none of these differences were statistically significant, it can be supposed that antenatally, in the prAEDF group, lower fetal GA was probably associated with a more “toxic” intrauterine medium compared with the trAEDF group. After delivery, the only parameter was prematurity, which was also higher in the prAEDF group.

The rate of worsening of the general maternal condition in the prAEDF group was significantly higher than that in the trAEDF group. This was especially supported by the fact that they had more than twice the rate of patients with therapy-resistant hypertension. However, why this difference was noticed only in cardiovascular and not in hepatic, renal, ocular, and brain territories is very difficult to explain. We assumed that the lack of cases with placental abruption and the relatively low incidence of retinal detachment and/or HELLP syndrome in the prAEDF group were probably consequences of a shorter maternal exposure to pathophysiological conditions, due to the shorter period until delivery imposed by the fetal and/or maternal condition. No maternal death resulted after permanent and careful monitoring of patients.

It can be easily concluded that the lower GA(LMP) at delivery, and consecutively, the lower fetal weight at birth in the prAEDF group compared with the trAEDF group, were the result of the earlier termination of pregnancy imposed on patients due to the alteration of either fetal and/or maternal condition.

We considered that a “pure” AEDF in the UA of restricted fetuses, in the absence of other complications, was not an indication for immediate fetal delivery. Arduini [43] reported a possible postponement of delivery in such cases for as long as 26 days. In the present study, the maximum number of days “gained” from diagnosis until delivery was 11, noticed in two cases of 27-week and 31-week pregnancies, respectively. The right moment for the delivery of these fetuses has always been a great challenge for obstetricians, who have to choose between keeping the fetus in a highly inappropriate environment, while maintaining an increased specific maternal risk induced by ESP and the delivery of an extremely premature fetus (with serious consequences).

Our choosing of C-section for all live restricted fetuses before 34 weeks GA was partially concordant with Alanis [44], who found no improvement in outcome of “healthy” fetuses between 28 and 34 weeks of amenorrhoea by C-section in mothers with EPS, but who recommended the C-section in GRFs. C-section was performed at a similar frequency in both groups, although the rates of either fetal deterioration or worsening maternal condition were higher in the prAEDF group. This apparent paradox was the result of an increased rate of spontaneous labour in the trAEDF group, which was followed by a C-section in fetal interest. The longer period from diagnosis to delivery was probably the motif for the increased (but not statistically significant) rate of spontaneous labour.

Another important finding in this study was the worrisome occurrence of EPS in the younger population (22.4 ± 5.9 years) compared with similar reports [44,45], which was probably due to regional social and demographic characteristics.

An important limiting factor of this study was the relatively small size of the group included. This was due to the very low incidence of ESP with fetuses revealing growth restriction and AEDF in the UA, registered in our three tertiary medical units, which was around 4/10.000 pregnancies. However, the rarity of these cases made the subject so attractive and, probably, future reports, eventually on larger cohorts, will provide new information on this domain.

## Conclusions

AEDF in the UA during EPS is a serious condition, and it is highly recommended that such cases be monitored in tertiary-level hospitals. Complex fetal Doppler evaluation of the UA, MCA, and DV, together with multidisciplinary maternofetal monitoring, is compulsory for the follow-up of these pregnancies and especially for the right decision on the moment of delivery.

The lack of effect of steroids was probably a sign of a more severe vascular dysfunction that was responsible, on the maternal side, for earlier symptoms and earlier diagnosis of EPS, and on the fetal side, for an increased rate of fetal hypoxia. Therefore, the steroid effect on AEDF in the UA can be a useful prognostic factor for worsening general maternal condition, especially with regard to non-responsive arterial hypertension or acute fetal deterioration.

Furthermore, the Dex no-effect was correlated with poor neonatal status at birth and neonatal complications with hypoxic etiology, such as RDS and IVH, but not with perinatal mortality. It is highly likely that larger cohorts will also confirm the correlation between the effect of Dex and fetal acidosis or PVL.

Finally, we consider that, in addition to the prognostic value, the sensitivity of AEDF in the UA to steroids, in such cases, should be a compulsory subject during antenatal parental counselling for a more accurate assessment of possible perinatal and maternal risks.

## Data Availability

The authors confirm that the data supporting the findings of this study are available within the article [and/or] its supplementary materials.
